# Traumatism of the Intestines

**Published:** 1906-11

**Authors:** Herman E. Hayd

**Affiliations:** Buffalo, N. Y.; Surgeon to the German and the German Deaconess’s Hospitals


### CLINICAL REPORT.

### Traumatism of The Intestines.

By HERMAN E. HAYD, M. D., Buffalo, N. Y.
Surgeon to the German and the German Deaconess's Hospitals.

A rare and interesting case occurred recently in my service at the German Hospital. A man, a Pole, 52 years of age, was injured by a plank which fell upon his abdomen at 7 o'clock in the morning. He was seen by Dr. Schroeter, who made the diagnosis of a ruptured bowel and sent the man to the hospital. At 2 p. m., I saw the patient and concurred in the diagnosis and immediately performed an abdominal section. There were no external marks or other evidences of violence. The abdomen was distended and extremely tender, while there was a general board-like hardness and rigidity of the muscles; temperature 100, pulse 128. The man was in great agony. There had been no vomiting and he had passed no gas.

Upon opening the peritoneal cavity, free fluid ran out mixed with pieces of carrot and potato, which had been eaten for supper the previous night. The bowels were quickly searched and a rent one inch in length was found in the jejunum. The edges were sewed together with through-and-through catgut suture and reinforced with silk Lembert. The peritoneal cavity was freely irrigated with warm salt solution and an abundance of carrot, potato, and undigested food was washed out; when the return fluid became reasonably clear the wound was quickly closed, after placing two drainage tubes well down into the pelvic cavity. The patient was put to bed in the elevated Fowler position and salt solution was slowly and continuously injected into the rectum, according to the Murphy method.

In a paper read by Dr. John B. Murphy, of Chicago, at the Cincinnati meeting of the American Association of Obstetricians and Gynecologists, September, 1906, on the treatment of acute diffuse peritonitis from perforative appendicitis, he reported 36 cases with 35 recoveries.

He makes a small incision and removes the appendix with as little shock, trauma, and injury to the peritoneum as possible. A drainage tube is placed in the pelvis and the wound closed without irrigation, no matter how foul the peritoneal contents are. The patient is put to bed in the elevated Fowler position and a normal salt solution is continuously injected into the rectum through an ordinary douche tip from a fountain syringe, elevated four or five inches above the level of the nates, at the rate of one and one-half pints per hour so that thirty pints are instilled in the twenty-four hours. Care must be exercised in placing the fountain syringe so that the fluid will run slowly and all be absorbed in the rectum. Perhaps it may be necessary to elevate or lower the bag one or two inches, in case the flow is too slow or too fast.

By this method of treatment the peritoneal cavity is made, if possible, a secreting membrane instead of an absorbing one as its bloodvessels are overfilled with fluid; secondly, the blood is washed of its toxins; and thirdly, the patient is given the best chance to develop a reactive leukocytosis. Murphy maintains that by washing the bowels and irrigating the peritoneal cavity the endothelium is removed from the peritoneal surfaces, increased absorption of toxins results and, as a consequence, death takes place before reaction can be brought about.

In my case I irrigated because there was so much foreign gross material in the peritoneal cavity, which had passed out of the rent in the bowels. The patient left the operating table in excellent condition and had a fair night. On the following evening he began to fail, his kidneys only excreted ten ounces of urine in the twenty-four hours, and he died on the following morning, thirty-six hours after the operation.

The case is instructive in showing that a rupture of the abdominal viscera can take place from external violence and yet there may be no visible evidences of injury or penetration of the abdominal wall. If this patient had been operated immediately upon the receipt of the injury or a short time afterward, he probably would have recovered.

493 Delaware Avenue.

